# Speech Databases of Typical Children and Children with SLI

**DOI:** 10.1371/journal.pone.0150365

**Published:** 2016-03-10

**Authors:** Pavel Grill, Jana Tučková

**Affiliations:** Department of Circuit Theory, Czech Technical University in Prague, Faculty of Electrical Engineering, Prague, Czech Republic; Birkbeck College, UNITED KINGDOM

## Abstract

The extent of research on children’s speech in general and on disordered speech specifically is very limited. In this article, we describe the process of creating databases of children’s speech and the possibilities for using such databases, which have been created by the LANNA research group in the Faculty of Electrical Engineering at Czech Technical University in Prague. These databases have been principally compiled for medical research but also for use in other areas, such as linguistics. Two databases were recorded: one for healthy children’s speech (recorded in kindergarten and in the first level of elementary school) and the other for pathological speech of children with a Specific Language Impairment (recorded at a surgery of speech and language therapists and at the hospital). Both databases were sub-divided according to specific demands of medical research. Their utilization can be exoteric, specifically for linguistic research and pedagogical use as well as for studies of speech-signal processing.

## Introduction

Speech signal analysis is divided into many areas, such as the identification of speakers and speech synthesis as well as subsidiary methods for medical diagnosis (pathological speech). One important component of these speech operations is the existence of corpora and specialized databases. Various aspects of speech operations demand different corpora contents and methods of database processing. Interests in speech processes can be focused on profession, gender, age, or, for example, on type of speech disorder.

One of the older corpora consisted of isolated words (i.e., digits). A paper [1] described a large isolated English digit database that was designed for the training and evaluation of statistical algorithms and neural networks (1108 speakers were recorded). In the Faculty of Applied Sciences of the University of West Bohemia in Pilsen, corpora have been created that are intended for the development of systems for speech recognition and speech synthesis [2]. Other Czech corpora have been recorded in a special environment (e.g., in a car) [3]. Czech corpora also include the Telephone Speech Data Collection for Czech and the Czech SpeechDat-E Database (Eastern European Speech Databases for Creation of Voice-Driven Teleservices), created at the Faculty of Electrical Engineering and Communication of the Brno University of Technology and at the Department of Circuit Theory in the Faculty of Electrical Engineering of CTU in Prague [4]. Most corpora contain utterances of adult men and women (for more details on corpora, see Psutka et al. [2]). Among foreign corpora, the TIMIT (Acoustic-Phonetic Continuous Speech Corpus) is used very frequently. It is a corpus of read speech for acoustic-phonetic studies and for the development and evaluation of automatic speech recognition systems that contains recordings of 630 speakers of eight major dialects of American English [5]. We encounter corpora or specific databases of some types of speech, such as children’s speech or pathological speech for example, less frequently. In the Voice Disorders Database Learning Resource for Researchers and Clinicians [6], speech-language pathologists at the Massachusetts Eye and Ear Infirmary (MEEI) in Boston and at Kay Elemetrics Corporation in Pine Brook, NJ have teamed up to produce the first clinical database of more than 1,400 voice samples for speech-language pathologists and voice scientists in the United States. A paper by Kim et al. [7] described a database of dysarthric speech produced by 19 speakers with cerebral palsy. This database provides a fundamental resource for automatic speech recognition development for people with neuromotor disabilities. Saz et al. [8, 9] described the corpus of disordered speech (comprising different speech disorders) from 14 young impaired speakers, including 7 males and 7 females aged 11 to 21 years, which was used for automatic speech recognition (ASR). The databases described by Miller et al. [10] were built using recordings from children aged 5 to 18 years. One database was recorded based on telephone quality (25 words or phrases) and the other using high-fidelity recording equipment (20 words, 5 sentences). IPA symbols were applied for transcription.

The corpora (databases) created at the LANNA (Laboratory of Artificial Neural Network Applications [11]) in the Department of Circuit Theory of the FEE CTU in Prague are specialized and intended mostly for medical research, but are useful in the field of linguistics as well. The stimulus for their creation came from cooperation with the Department of Paediatric Neurology at the 2nd Faculty of Medicine of the Charles University in Prague, which is supported by several grants from the IGA MZ CR (Science Foundation of the Ministry of Health of the Czech Republic). This research focuses on Specific Language Impairment (SLI), also termed Developmental Dysphasia (DD); the term DD is used primarily by pediatric neurologists. One of the symptoms leading to a diagnosis of DD is speech impediment. Because no speech corpora for this specific health problem existed, it was necessary to create one, and we describe these corpora in the following article.

## Methods

### Ethics Statement

This study was conducted in the Department of Circuit Theory of the Faculty of Electrical Engineering of the CTU in Prague. The Ethics Committee of Motol University Hospital in Prague, Czech Republic approved the research. All the parents provided written informed consent on behalf of their children prior to participation in the study.

### General Characteristics of the Recorded Speech

The first step in creating the corpus was the selection of words for speech recording. One of the criteria for inclusion in the study involved a psychological examination of the children. Considering the specific conditions under which the recordings were made (i.e., background noise, behavior of the children during the recording, etc.), it was necessary to use nonstandard methods of computerized processing of the speech. Due to having longtime experience with the application of artificial neural networks (ANN) [12], our research team decided to use these networks for the analysis of the speech of children with SLI (hereafter referred to as “cases”), as well. We wanted to make use of the strong attributes of these networks such as their robustness, i.e., their ability to process recordings even with high surrounding noise levels. We decided to apply Self-Organizing Maps (SOM) in our research. In addition to the children's utterances, it was necessary to record a comparative set of utterances from children without any disorders (hereafter referred to as “controls”). In the following text, we will not adhere strictly to the division between corpora and databases; such a distinction is solely analytic, and formal criteria must be amenable to immediate demands.

The size of the individual databases (i.e., the amount of collected data) was a major problem in this study. The number of recorded children enrolled was dependent on the parents’ informed consent. This was difficult to obtain, especially for the controls. Additionally, the number of children with a speech disorder in the population (particularly with SLI) is relatively small; developmental dysphasia affects about five to seven percent of the pediatric population [13, 14].

All recordings, data and applications are saved on the server of the LANNA research group and are available there to authorized users or on the server of The LINDAT/CLARIN Centre for Language Research Infrastructure. Anyone interested in speech recordings can contact the head of the LANNA research group by web address (http://ajatubar.feld.cvut.cz/lanna/) or by email (tuckova@feld.cvut.cz and grillpavel@gmail.com). Additionally, these data sets and software can be downloaded from the website of the LINDAT/CLARIN group (http://hdl.handle.net/11372/LRT-1597).

### Psychological Examination

The children included in this research had to be examined by a clinical psychologist. The examination took place in the Department of Pediatric Neurology at the 2nd Faculty of Medicine of Charles University in Prague. The parents were present during the examination, which took one day. The children did not take any medications. The following tests were used during the examination:

Gesell Developmental Diagnosis [15]
Stanford-Binet Intelligence Test, Fourth Edition
Additional tests standardized for the Czech language: sound differentiation test, world differentiation test, auditory analysis and synthesis test
Special test of perceptual skills, graphomotor skills and visuomotor coordination
Spontaneous talking evaluation
Figure drawing and tracing.

The inclusion criteria used were as follows: performance intelligence quotient (PIQ) ≥ 70, disturbed phonemic discrimination, disturbed language on various levels–phonologic, syntactic, lexical, semantic and pragmatic [16].

Participants were assessed by other specialists as well. Neurological examinations showed no abnormalities. Motor milestones were within normal range. No participants had a hearing impairment or were receiving antiepileptic medication. No child had been diagnosed with a pervasive developmental disorder or other dominating behavioral problems. Additionally, none of the children had a history of language or other cognitive regression [16].

The Stanford-Binet test [17] is one of the standard intelligence tests used in clinical pediatric psychology. The fourth revision of the test, published in the Czech Republic in 1995, covers ages 2 to 14 years. In this revision, the test focuses on four areas of evaluation: verbal reasoning, abstract-visual reasoning, quantitative reasoning and short-term memory. The advantage of the fourth revision is the possibility for a relatively reliable evaluation profile and for a comparison of the levels of the individual skills of the child. Furthermore, the different parts of the test can be used separately. The primary goal of the test is to diagnose children with general learning difficulties and distinguish these general learning difficulties from specific learning disabilities.

The terms “special test of perceptual skills”, “graphomotor skills” and “visuomotor coordination” ([13, 14, 18] and [19]) cover the set of psychomotor activities that children exercise when writing and drawing. These activities focus not only on the coordination and movements of the arms, hands and fingers while grasping writing tools (and manipulating them) but also on constructive skills such as various puzzles. Writing and drawing are not associated with the motoric cortex alone; they are influenced by other centers located in different areas of brain, and the reverse is also true. Due to these complex influences, an assessment of graphomotor skills can aid in the diagnosis of neurological disorders. These skills influence speech and language mechanisms as well, which is why they are part of the examination performed to reveal developmental dysphasia.

Specific Language Impairment, or Developmental Dysphasia, ([13], [14], [20], [21], [22]) is diagnosed when a child has delayed or disordered language development for no apparent reason. It is described as a language disorder that delays the mastery of language skills in children who have no hearing loss or other developmental delays. These children fail to acquire their native language properly/completely, despite having normal non-verbal intelligence, no hearing problems, and no known neurological dysfunctions or behavioral, emotional or social problems [23]. It is estimated that SLI affects approximately 5–7% of the kindergarten population [24]. It has been demonstrated in various heritability studies, such as genetic etiology studies, family evaluations and studies of twins [25], that SLI includes a quite significant genetic component. Another study showed that SLI affects boys much more frequently than girls [24]. The manifestations of the impairments in SLI are mainly reduced vocabulary development at early ages and, typically, difficulty in manipulating linguistic rules of inflection and derivation. This leads to use of incorrect syntactic structures in a patient’s native tongue. Usually, the language comprehension of patients is better than their own language production. Children with SLI may have difficulties in non-linguistic cognitive skills, e.g., executive functions, mental rotation, or motor ability [26]. Other difficulties occur, particularly in other cognitive domains such as working memory [27], [28], and these difficulties may be associated with reading impairments [29], [30].

Numerous studies have addressed the question of what the underlying problem is that is causing the observed language difficulties. In these studies are solved directly theories of language acquisition and language representation and processing [31], [32]. Three of the most frequent hypotheses for the causes of SLI are as follows:

1. Children with SLI possess a general processing deficit reflected in their slower linguistic processing but have relatively normal linguistic representations [31], [32].
2. Children with SLI have a developmental delay in language acquisition. They have normal linguistic and other cognitive abilities but later timing in the triggering or onset of language acquisition processes [26].
3. Children with SLI have relatively intact cognitive abilities, but their difficulties are with grammar or specific subcomponents of grammar [33], [34].

This impacts of this disorder on children in everyday life mean that an affected child does not have the same speech skills as other children of the same age because the affected child’s speech skills are delayed. Children with SLI thus experience a type of social barrier that separates them from their peers and disrupts their social lives.

### Speaking Tasks

Before recording the children's speech, it was necessary to select suitable words, phrases and sentences. It was essential to select utterances that children of a given age will be able to articulate. Our research teams worked with children aged from 4 to 11 years. Younger children cannot yet read and must therefore repeat spoken utterances, so it was necessary to maintain the same conditions for all enrolled children. The main criteria used in creating the corpus were the selection of a suitable text and the selection of participants for speech recording. Upon selection of suitable utterances, clinical psychologists cooperated with speech and language therapists. The words and phrases were directly selected for research among children with this disorder (specific language impairments), and they were selected while accounting for the physiological and mental development of the children. The specific utterances that comprise the speech database are divided into 13 parts (Table 1).

**Table 1:** Speech database–structure and types of utterances used in our research.

Task code	Type of part	# Patterns	Descripton	
**[T 1]**	Vowels	5	Czech	"a", "o", "u", "e", "i"
** **			English	"a", "o", "u", "e", "i"
**[T 2]**	Consonants	10	Czech	"m", "b", "t", "d", "r", "l", "k", "g", "h", "ch"
** **			English	"m", "b", "t", "d", "r", "l", "k", "g", "h", "ch"
**[T 3]**	Syllables	9	Czech	"pe", "la", "vla", "pro", "bě", "nos", "ber", "krk", "prst"
** **			English	"pe", "la", "vla", "for", "bě", "nose", "take", "neck", "finger"
**[T 4]**	Two-syllable words	5	Czech	"kolo", "pivo", "sokol", "papír", "trdlo"
** **			English	"wheel", "beer", "falcon", "paper", "boob"
**[T 5]**	Three-syllable words	4	Czech	"dědeček", "pohádka", "pokémon", "květina"
** **			English	"grandfather", "fairy tale", "Pokemon", "flower"
**[T 6]**	Four-syllable words	3	Czech	"motovidlo", "televize", "popelnice"
** **			English	"niddy noddy", "television", "dustbin"
**[T 7]**	Difficult words	2	Czech	"různobarevný", "mateřídouška"
** **			English	"varicoloured", "thyme"
**[T 8]**	Geminate words	3	Czech	"pohádková víla", "kouzelný měšec", "čarotvorný hrnec"
** **			English	"fairy", "magic pouch", "magic pot"
**[T 9]**	Accretion of range of words	4	Czech	"voda", "živá voda", "živá a mrtvá voda", "pramen s živou a mrtvou vodou"
** **			English	"water", "live water", "live and dead water", "source of live and dead water"
**[T 10]**	Sentence	1	Czech	"Když šla červená Karkulka k babičce, potkala zlého vlka."
** **			English	"When Little Red Riding Hood went to her grandmother, she met bad wolf."
**[T 11]**	Auditory differentiation	10	Czech	"pes—nes", "ten—den", "kůl—vůl", "hrát—brát", "ječí—ježí", "ble—ple", "kloč—kloč", "kvěš—kveš", "šný—šní", "vošl—vočl"
** **			English	Change in one phoneme in the word. For example: "pes—nes", …
**[T 13]**	Describe the picture	1	English	"Look at the laughable clown."—A spontaneous description of the girl's picture.

### Speech Database and Participants

In this section, we will describe the creation and contents of the individual databases and the information about the participants that was compiled in cooperation with medical researchers working on conjoined grants.

The entire database contains three subgroups of recordings of children's speech from different types of speakers. The first subgroup (controls, or H-CH) consists of recordings of children without speech disorders; the second subgroup (cases, or SLI-CH I) consists of recordings of children with SLI; the third subgroup (cases, or SLI-CH II) consists of recordings of children who have SLI of different degrees of severity (1 –mild, 2 –moderate, or 3 –severe). The speech and language therapists and specialists from Motol Hospital decided upon this classification. The children’s speech was recorded during the period 2003–2013. The database has two specific components. The first component is the recording database. These databases were commonly created in a schoolroom or a speech and language therapist’s consulting room in the presence of surrounding background noise. This situation simulates the natural environment in which the children live and is important for capturing the normal behavior of the children. The second component consists more of recordings of individual children. Using this approach, we are able to compare the course of therapy.

### H-CH database

The database of controls was created as a referential database for the computer processing of children’s speech. The children’s speech is marked when minor speech errors are evident (e.g., incorrect articulation of sibilants, “r” and “ř”), or when it was the speech of children visiting a speech and language therapist (“controls with defect”). The speech recordings were recorded in the first four grades of elementary school and in preschool. The quality of these speech signals (due to outside noises) makes the analysis more difficult and may influence the results, especially when using standard methods of computerized speech processing. This problem was eliminated by the application of an ANN (artificial neural network). The H-CH database contains a total of 70 native Czech participants (25 boys, 45 girls) without speech defects and 33 native Czech participants (17 boys, 16 girls) with speech defects, all aged 4 to 10 years. The speech recordings were recorded from 2003 to 2005. The description of the database structure of the control database is provided in Table 2.

**Table 2:** Description of All Databases. Subgroup H-CH is for controls and subgroups SLI-CH I and SLI-CH II are for cases.

	H-CH		SLI-CH I	SLI-CH II
		WITH DEFECT		
**Girls**	45	16	13	26
**Number of recordings**	45	16	22	45
**Boys**	25	17	33	46
**Number of recordings**	25	17	64	88
**All children**	70	33	46	72
**All recordings**	70	33	86	133
**All utterances**	4620	2178	5676	8819

### SLI-CH I database

This database contains the recordings of the cases (pediatric patients with SLI). Trends in the way that speech changed during the given time period (approximately 3 months) were the determining factors for inclusion in this database, rather than the degree of severity of the children’s diagnosis. The SLI-CH I database contains a total of 46 native Czech participants (33 boys, 13 girls) aged 4 to 12 years and was recorded during the period 2005–2008. The description of the SLI-CH I database is provided in the third column of Table 2.

### SLI-CH II database

This database was recorded in a speech and language therapist’s office [35, 36]. For the purposes of the research study, the children were separated into three groups depending upon the severity of their diagnosis (mild, moderate, or severe). The SLI-CH II database contains a total of 67 native Czech participants (44 boys, 23 girls) aged 6 to 12 years and was recorded during the period 2009–2013. The lower age limit had to be raised due to the need for an MR tractography examination [37]. The recordings of some children were repeated after several months. The description of the SLI-CH II database is provided in the fourth column of Table 2.

## Hardware and Software

The H-CH database was recorded on the SONY digital Dictaphone (sampling frequency, fs = 16 kHz, 16-bit resolution in stereo mode in the standardized wav format) and on the MD SONY MZ-N710 (sampling frequency, fs = 44.1 kHz, 16-bit resolution in stereo mode in the standardized wav format). The corpus was recorded in the natural environment of a schoolroom and in a clinic.

The SLI-CH I database was recorded in a doctor´s office in the faculty hospital, whereas the SLI-CH II database was recorded in a private speech and language therapist’s office. The recordings contain background noise. Another problem encountered was the age of the children: preschool children cannot read, and text that is read is processed by a different part of the brain than text repeated in spoken words in imitation of the assistant. The speakers were recorded using a lapel microphone (which presents a problem with maintaining attention). The sound recordings were saved in the standardized wav format. The sampling frequency was set to 44.1 kHz with 16-bit resolution in mono mode.

Two software packages were created to simplify the creation and management of our speech database, and these were designed to be used by researchers only.

### Create Database Structure (CDS)

The program for automatic creation of the database structure was developed in the MATLAB [38] environment. Some of the parameters that were included are as follows: Name of child, Surname of child, Abbreviation of child, Date of the recording and Number of recordings. The button entitled “Create a directory” was used for the selection of the working directory in which the directory structure for the child was created.

### Web Administration (WA)

Another application that was created is an online administration of the developed SLI-CH II database. This web application [39] is accessed via a login window. The administrator assigns a name and password, and the login is secured by a cryptographic hash function of the SHA-1 type [40]. This online application was created to provide immediate access to the data records of the children and to maintain the ability to create the text that the children repeated for the recordings. We found that cases were able to remember this text. It is possible to choose the order of recorded speeches (for repeated recordings), and the application is able to generate three variants of text:

Original word order
Random word order within sub-groups
Random order of all words

This application saves the history of the recordings, which includes some supplementary information. The history contains information about the participant with SLI (name, surname, date of birth, diagnosis and its degree, and other supplementary information) and detailed information about the recordings (number of recordings, serial number, date of recording, type of recorded text, list of all speeches, etc.). This complete information is able to be viewed immediately, edited or saved as.txt files by all researchers involved. All the information is available only to appointed medical staff or researchers.

### Processing of the Recordings and the Software Used

Before processing the recordings captured on a portable digital recording device (minidisc, Dictaphone, etc.), it was necessary to unify the parameters with the recordings captured on the computer. The sound intensity for all the recordings had to be brought to the same level, and the two-channel recordings (stereo) had to be converted to one-channel (mono). The remainder of the processing was the same for all the recordings. The program COOL EDIT PRO 2 [41] (now known as Adobe Audition) was used for processing the recordings.

This commercial software, which is often used in the professional sphere, was employed for sophisticated work in digital audio formats. The children's speech was separated from that of the therapist by the LABELING program [35], which segments the speech signal. This program is part of the SOMLab program system [42], which was developed in the LANNA [11] at CTU FEL in Prague and was built using the MATLAB script language on the base SOM Toolbox (see paragraph 4.4). The program is available free of charge under the General Public License (GNU) from the SOM Laboratory for Speech Analysis [43]. For projects regarding speech, new special M-files, which should be part of the supporting program package, were created. All the cases and controls were labeled by specific individuals.

### Speech Analysis Software

The software called SOM Toolbox was applied in our experiments. SOM Toolbox was developed in the Laboratory of Information and Computer Science (CIS) at the Helsinki University of Technology and was built using the MATLAB script language. SOM Toolbox contains functions for the creation, visualization and analysis of Self-Organizing Maps. The Toolbox is available free of charge under the General Public License [43].

The original software FORANA [44], [45] was developed for formants analysis. It is based in the MATLAB programming environment. The development of this software was mainly driven by the need to have the ability to correctly complete the formant analysis. Usually, the extraction of formant frequencies from speech signals was performed using the PRAAT [46] acoustic analysis software. However, because the use of the PRAAT software produced formant classification errors in the course of the analysis (for higher formants), the results obtained using this approach could not be considered relevant. Another factor taken into consideration in designing the FORANA software was the need for full automation of the process of extracting formants from the recorded speech signals.

### Open SMILE

The Munich open toolkit Speech and Music Interpretation by Large Space Extraction (openSMILE) [47] is a modular and flexible feature extractor for signal processing and machine learning applications. The primary focus is clearly on audio-signal features. It is written purely in the C++ language and runs on various mainstream platforms such as Linux, Windows, and MacOS. OpenSMILE is designed for real-time online processing but can also be used online in batch mode for the processing of large datasets.

### Supervised Self-Organizing Maps as Data Classifiers

The program package called the SOM Laboratory (SOMLab) was used for one part of the automatic speech analysis. It is based on the Self-Organizing Map (SOM) [42] application for speech signal processing in the Laboratory of Artificial Neural Networks Application (LANNA) [11]. The program takes advantage of label information from input data and neural networks. SOMLab is a complex system that was created as a user-friendly application of the artificial neural networks in speech analysis. The program package consists of the tools necessary for utilizing the SOMs. Individual tools can be divided into three categories: pre-processing tools (tools for data preparation, data creation and analysis), processing tools (tools for methods of working with the SOMs, such as creating and training), and post-processing tools (tools for visualization, comparison and analysis of speech).

SOMLab works under the computational system known as MATLAB 7 and is compatible with Release 14 and higher. SOM Toolbox (see paragraph 4.4) was used for the creation of M-files in SOMLab. Some of its main advantages include the elimination of unsystematic errors during the manual data preparation process by the use of the automatic approach; the ability for exact selection of parameters (saving or loading them is useful for verification of the results); the unification of the analysis, which leads to greater ease in team research; and the fact that knowledge of the MATLAB script language or the SOM Toolbox functions is not necessary for working with SOMLab (further details about this software package have been described by Zetocha [42]).

## Applications and Results

In this section, we describe the current methods and associated results that we used for the speech diagnoses of cases (children with SLI), depending on the usage and processing of our speech database. The results from these methods represent one of the potential applications of this work for medical specialists to help in diagnosing these children.

For this research, 54 participants (35 boys, 19 girls) aged from 6 to 11 years were selected from the SLI-CH II subgroup (cases), and 44 participants (15 boys, 29 girls) aged from 6 to 10 years were selected from the H-CH subgroup (controls). All the results were computed at a significance level of p < 0.001, and all the research protocols were performed on both of these two selected subgroups.

### Formants and the Vocalic Triangle

**Fig 1:**
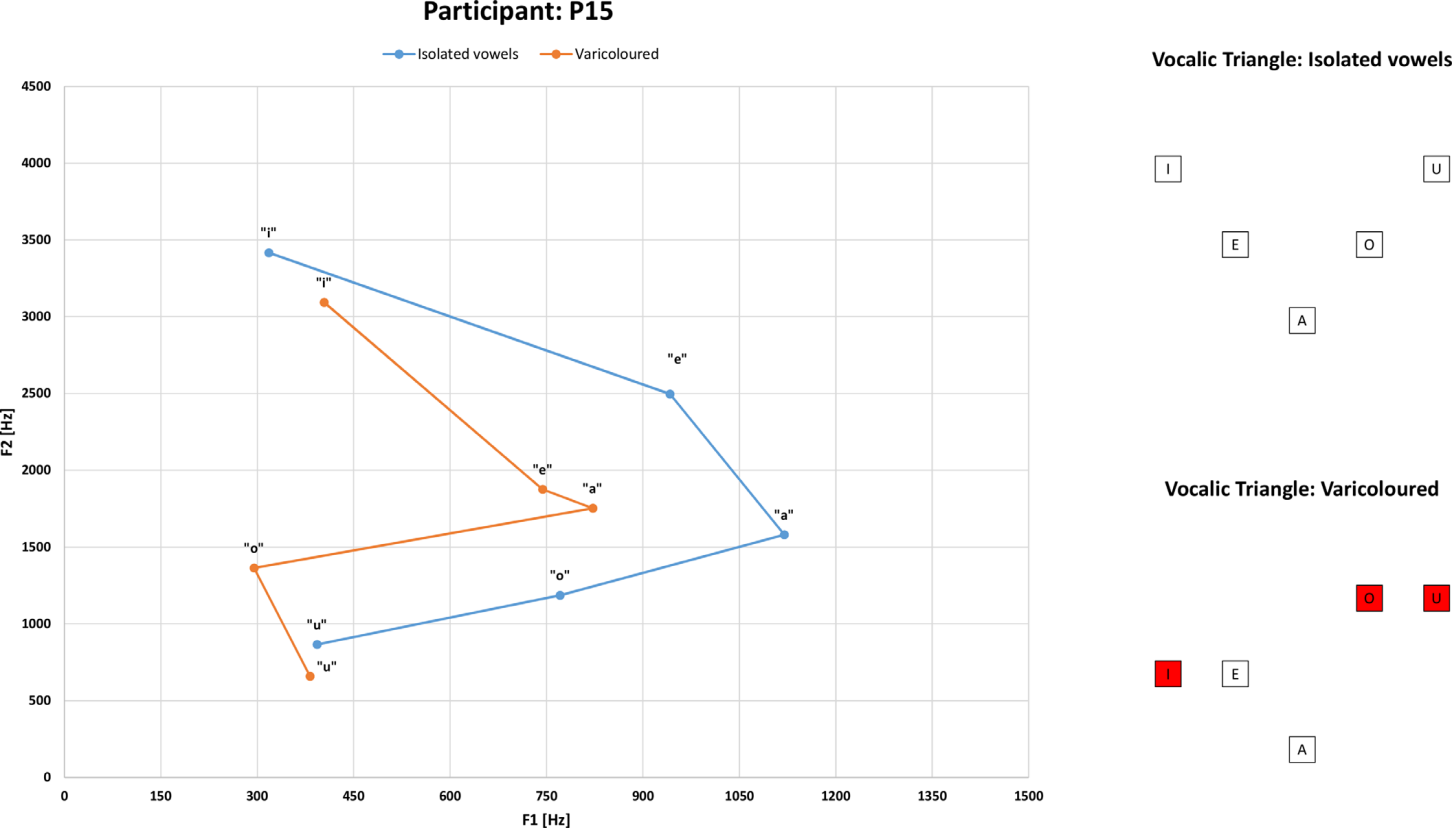
Comparison to different vocalic triangles. Blue is represents isolated vowels and orange represents vowels from the utterance “varicolored”. The color red represents vowels that are in the wrong place (graphs on the right side).

The ability to produce and perceive speech originates in certain parts of the human brain. SLI is described as a neurological disorder of the brain ([16, 19, 44, 48] and [49]). The formant is a parameter that possesses a physical dimension (the presence of acoustic energy across the spectrum of speech sounds). The existence of formants can be related to the activity of the human brain and movements taking place in a person’s articulatory system. This fact satisfies a condition for the use of formants in the classification of cases. One of the prerequisites for the use of formants as descriptive parameters is obtaining formant values with a minimum number of errors. For formant acquisition, we used the software tool FORANA (see paragraph 4.4 and references [44], [45]). Formants are usually defined as the spectral peaks of the sound spectrum of the voice (or the concentration of acoustic energy in the vicinity of a specific frequency). In the full range of the speech spectrum, we can count more formants. Changes to the speaker’s fundamental frequency (F0) are interpreted as a change in speech melody. Changes to the first formant (F1) correspond to changes in the vertical movement of the speaker’s tongue; changes to the second formant (F2) correspond to changes in the horizontal movement of the tongue; changes to the third formant (F3) correspond with actions taking place in the nasal cavity. The vocalic triangle describes the relationships between F1 and F2. The triangle divides individual vowels into three different categories, depending on the position of the given formant [50]. Particular vowels have clearly defined places in this vocalic triangle. The partial classification of SLI cases by the vocalic triangle is based on the comparison of two of the vocalic triangles (Fig 1). In this figure, the vocalic triangle representing isolated vowels is shown in blue, and the vocalic triangle representing vowels that have been extracted from the Czech word “různobarevný”(in English "varicolored") is shown in red. The vocalic triangle obtained when analyzing isolated vowels has the correct shape, but the triangle for the vowels contained in multisyllabic words or in the utterances has the wrong shape, and the vowels occupy a different place. More is discussed about this issue in [48]. This particular example can be used to demonstrate a relationship between the complexity of the words being spoken and the shift in the speech sound-frequency spectrum in cases. This finding can also be confirmed by listening to the given speech recordings.

### Tests of Utterances

Cases exhibit problems with auditory perception [17], especially in the areas of melody, tempo of speech, unit duration, rhythm, qualities of tone, auditory differentiation, memory, attention and word differentiation. The problems occur in the perception and processing of verbal stimuli, their storage in the memory and their recall, which includes memory learning. The problems also lie in the acoustic-verbal processes. The test of acoustic differentiation is based on the ability to distinguish the sounds of spoken language by hearing. In this case, the child's task is to distinguish between two syllables and recognize whether they are the same or not. Additionally, cases have a distinctly impaired ability to differentiate phonemes by hearing. They cannot distinguish acoustically similar words.

Our new method (more about this issue in a separate article) is based on a confusion matrix and the number of pronunciation errors made in utterances. We define the confusion matrix as a rectangular array comprising m rows and n columns, where m indicates the sounds from the word and n indicates the sounds from the utterance. Comparing the sounds from the word and the utterance, we arrive at the number of errors for one word and utterance (which indicates the sounds from the word), and we obtain the number of errors for one word. The number of errors is calculated as a penalty score in Eq 1:

PS=wp+up+mp (1)

where PS is the penalty score, wp is the number of wrong phonemes, up is the number of unspoken phonemes, and mp is the number of missing phonemes. The sum total, PS, indicates the total number of errors for the assessed child.

**Fig 2:**
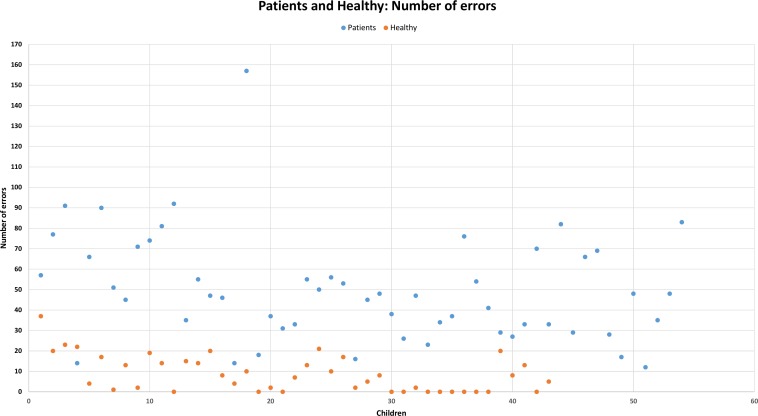
Number of errors for all participants. Blue represents cases, and orange represents controls. Samples with a higher number of errors are in a higher position.

The results of the analyses of errors, which represent all participants in our current study, are displayed in Fig 2. The X-axis represents all the children, and the y-axis represents the number of errors. The pronunciation errors of controls are displayed in orange, and those of cases are displayed in blue. A higher value in Fig 2 indicates a higher number of errors. Controls have a total number of errors in utterances that is much lower than that in cases. The methods (with their corresponding percent success rates) that were used for distinguishing these two groups are presented in Table 3. We achieved a 92.36% success rate for the best method. As it was necessary to determine the value of limits for every category, we used two different approaches to classify participants into these groups. In the first method, called HM (“hand-made”), we determined the value of the thresholds for each group. We used the minimum and maximum values from the controls and from the cases. Values located outside these limits were identified as a misclassifications. The other three methods (ANN methods) are based on artificial neural networks (ANN), and especially on self-organizing maps (SOM), which are a standard part of the Neural Network Toolbox in MATLAB. The differences between these ANN methods are developed through the setting of the initial weights in the training phase. In the first method, the weights are set on the original default value. The weights of the neurons are initialized to small random values. In the second method, the weights are set to the values of the thresholds (minimum and maximum values) of these groups. In the third method, the weights are set to the mean values of these groups, as shown in Fig 2 and Table 3.

**Table 3:** The percentage rate of the correct classification of the methods used for distinguishing between two groups. HM is for “hand-made” and ANN is for artificial neural networks.

Method	Percentage success rate
**HM**	65.44%
**ANN orig**	85.14%
**ANN min-max**	90.50%
**ANN mean**	92.36%

### Extracting Features for Recognition of Specific Language Impairments

To classify cases, formants and the number of errors in utterances were used in addition to low-level descriptors from the speech signal. As a suitable tool for obtaining these parameters, we employed the openSMILE toolkit [47] (freely available under the terms of the GNU General Public License). We chose 'emobase2010' as our reference set. The set contains a total of 1,582 features. The base contains 34 low-level descriptors (LLD) with 34 appended corresponding delta coefficients (see in Tables 4 and 5). On each of these 68 LDD coefficients, 21 functionals (1,428 features) were applied. In addition, 19 functionals were applied to the 4 pitch-based LLD and their four delta coefficient contours (152 features). Finally, the number of pitch onsets and the total duration of the input were appended (2 features). The names of the 34 low-level descriptors as they appear in the output file are shown in Table 4, and the names of the 21 functionals as they appear in the output file are shown in Table 5. The description was taken from the openSMILE toolkit tutorial [47], as shown in Tables 4 and 5.

**Table 4:** The names and descriptions of 34 low-level descriptors used for our experiments from the openSMILE toolkit [47].

Name	Number of coefficients	Description
**pcm_loudness**	1	Loudness as the normalized intensity raised to a power of 0.3.
**Mfcc**	15	Mel-Frequency cepstral coefficients 0–14.
**logMelFreqBand**	8	Logarithmic power of Mel-frequency bands 0–7 (distributed over a range from 0 to 8 kHz)
**lspFreq**	8	The 8 line spectral pair frequencies computed from 8 LPC coefficients.
**F0finEnv**	1	The envelope of the smoothed fundamental frequency contour.
**voicingFinalUnclipped**	1	The voicing probability of the final fundamental frequency candidate. Unclipped means that it was not set to zero when it falls below the voicing threshold.

**Table 5:** The names and descriptions of 21 functionals used for our experiments from the openSMILE toolkit [47].

Name	Description
**maxPos**	The absolute position of the maximum value (in frames).
**minPos**	The absolute position of the minimum value (in frames).
**amean**	The arithmetic mean.
**linregc1**	The slope (m) of a linear approximation of the contour.
**linregc2**	The offset (t) of a linear approximation of the contour.
**linregerrA**	The linear error computed as the difference of the linear approximation and the actual contour.
**linregerrQ**	The quadratic error computed as the difference of the linear approximation and the actual contour.
**stddev**	The standard deviation of the values in the contour.
**skewness**	The skewness (3rd order moment).
**kurtosis**	The kurtosis (4th order moment).
**quartile1**	The first quartile (25% percentile).
**quartile2**	The second quartile (50% percentile).
**quartile3**	The third quartile (75% percentile)
**iqr1-2**	The inter-quartile range: quartile2-quartile1.
**iqr2-3**	The inter-quartile range: quartile3-quartile2.
**iqr1-3**	The inter-quartile range: quartile3-quartile1.
**percentile1.0**	The outlier-robust minimum value of the contour, represented by the 1% percentile.
**percentile99.0**	The outlier-robust maximum value of the contour, represented by the 99% percentile.
**pctlrange0-1**	The outlier robust signal range 'max-min' represented by the range of the 1% and the 99% percentile.
**upleveltime75**	The percentage of time the signal is above (75% * range + min).
**upleveltime90**	The percentage of time the signal is above (90% * range + min).

A large number of features (in this case, 1,582 acoustic features) increases the ability to more accurately characterize the analyzed speech. On the other hand, it also increases the possibility that some of the data will be irrelevant or redundant. The procedure for feature selection was as follows: The data were evaluated using Spearman's rank correlation coefficient. All the data were compared using this correlation. The selected features were those that contained the best information about the internal dependencies of the data.

**Fig 3:**
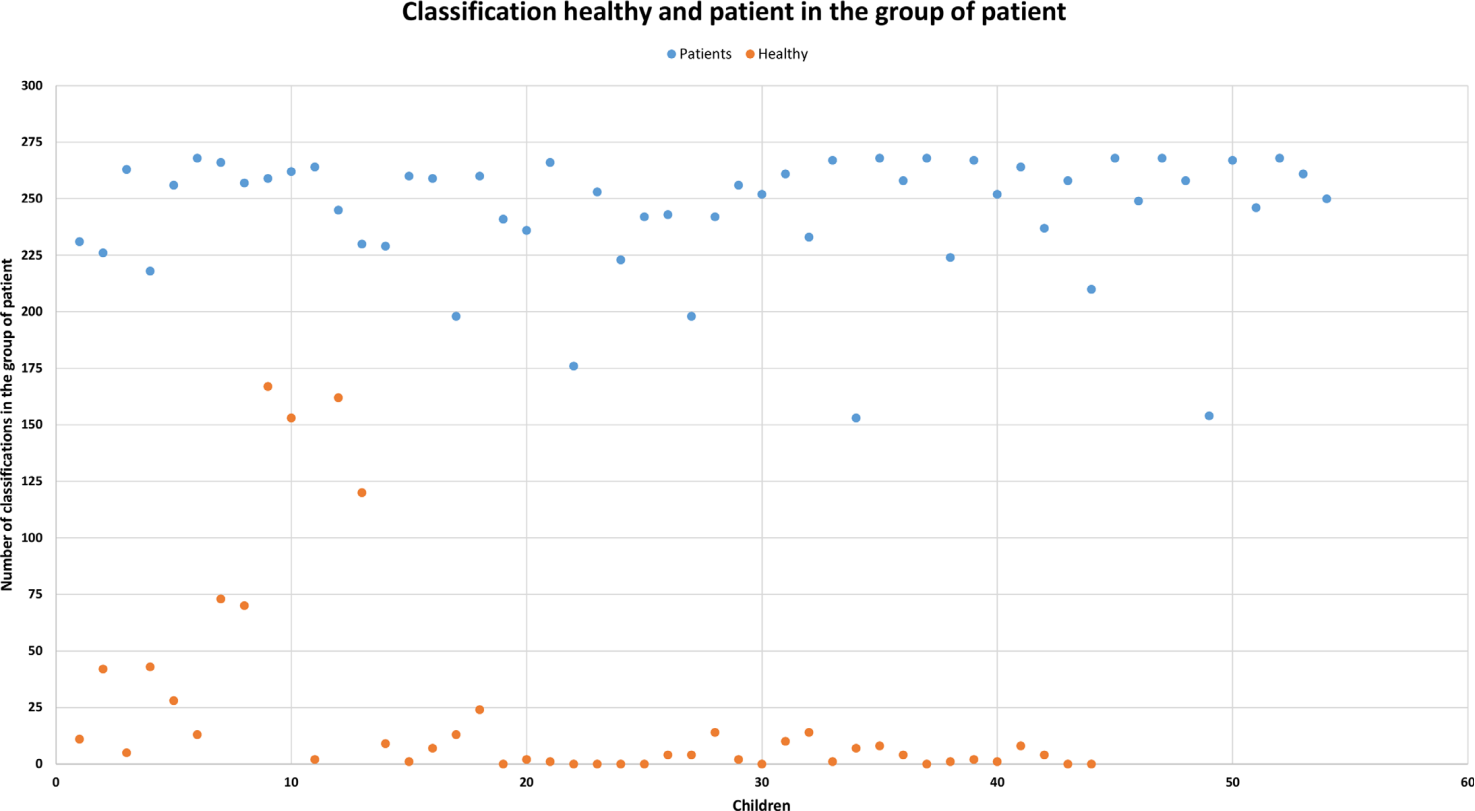
Number of classifications in the group of cases. Blue represents cases, and orange represents controls. Samples with a higher number of classifications are in a higher position.

**Fig 4:**
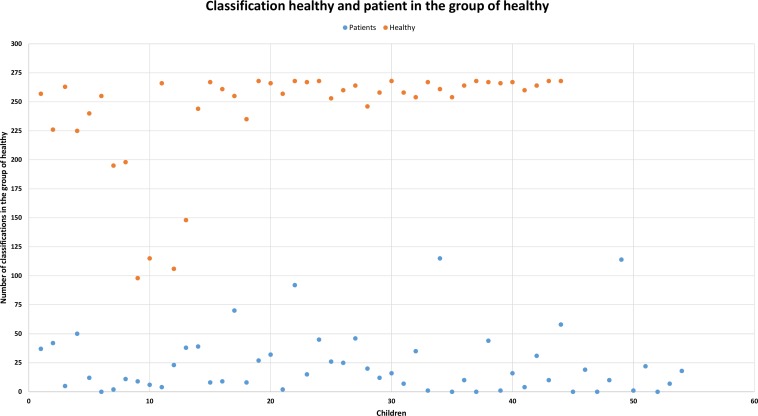
Number of classifications in the group of controls. Blue represents cases, and orange represents controls. Samples with a higher number of classifications are in a higher position.

We calculated the entirety of the features (1,582) for all the utterances (38) used in our research. We then determined the 30 best features (feature selection was performed using correlations) for all words and assigned a coefficient to each feature according to the group to which it was assigned (1 is for the correct group and 2 is for the incorrect group). We calculated the number of coefficients, and we classified the speaker in the group that had the best rank. This procedure was applied for each participant. In this manner we obtained a 96.94% success rate of correct classification of the speakers. The results of these analyses are displayed in Figs 3 and 4, which present all the speakers in our research study. The X-axis represents all the children, and the y-axis represents the number of classifications. In Fig 3, the aim is classification of both groups into the group of cases, and in Fig 4, the aim is classification into the group of controls. A higher number of classifications for a particular group assigned a speaker into that group. As displayed in the figures, speakers were almost always correctly determined. Values for controls are displayed in orange and values for cases are displayed in blue in Figs 3 and 4.

### A Database and Web Tool

**Fig 5:**
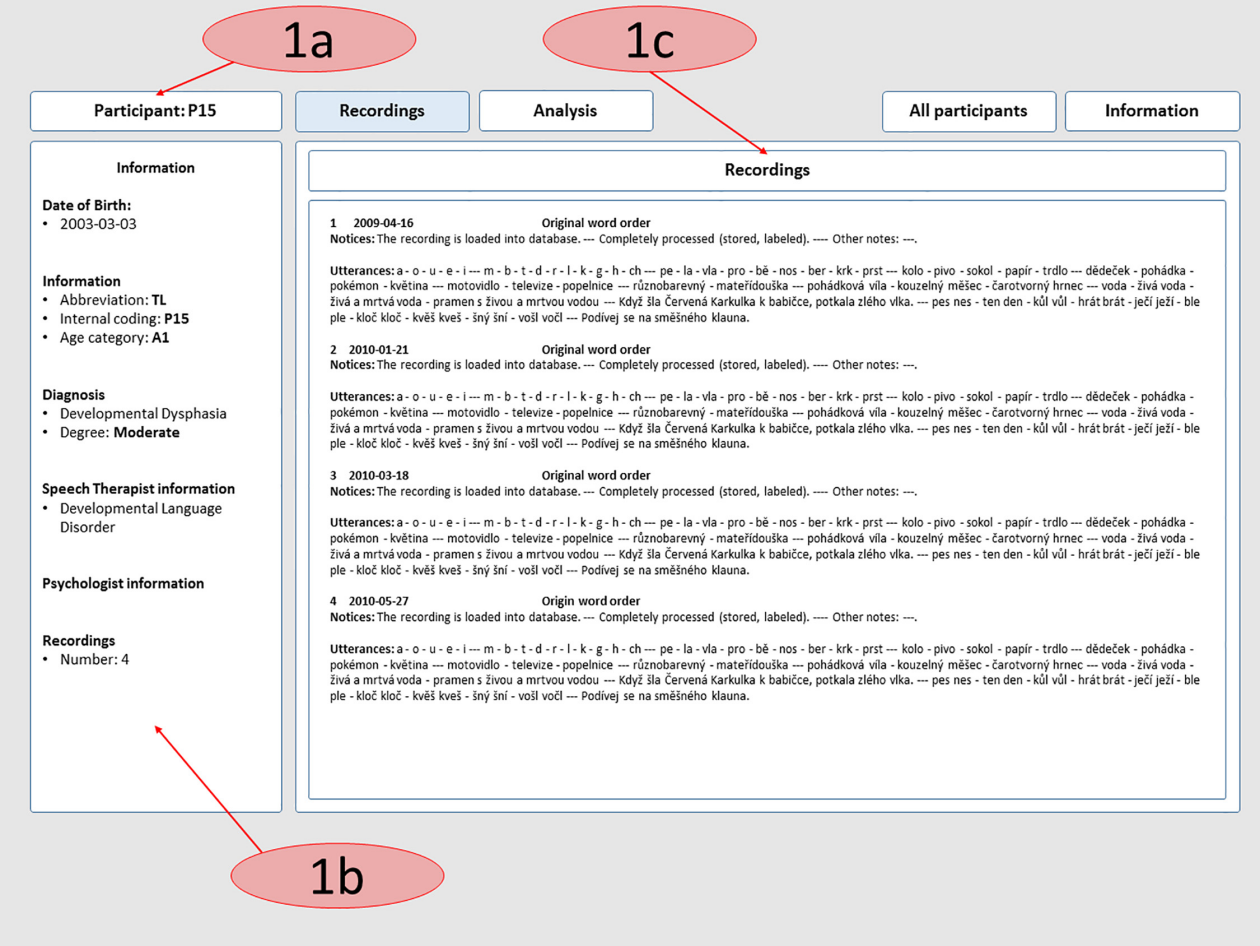
Overview of the core functionality of the web tool for medical staff. 1a) Name of the participant; 1b) All participant information; 1c) This panel is for the description and information of all recordings.

**Fig 6:**
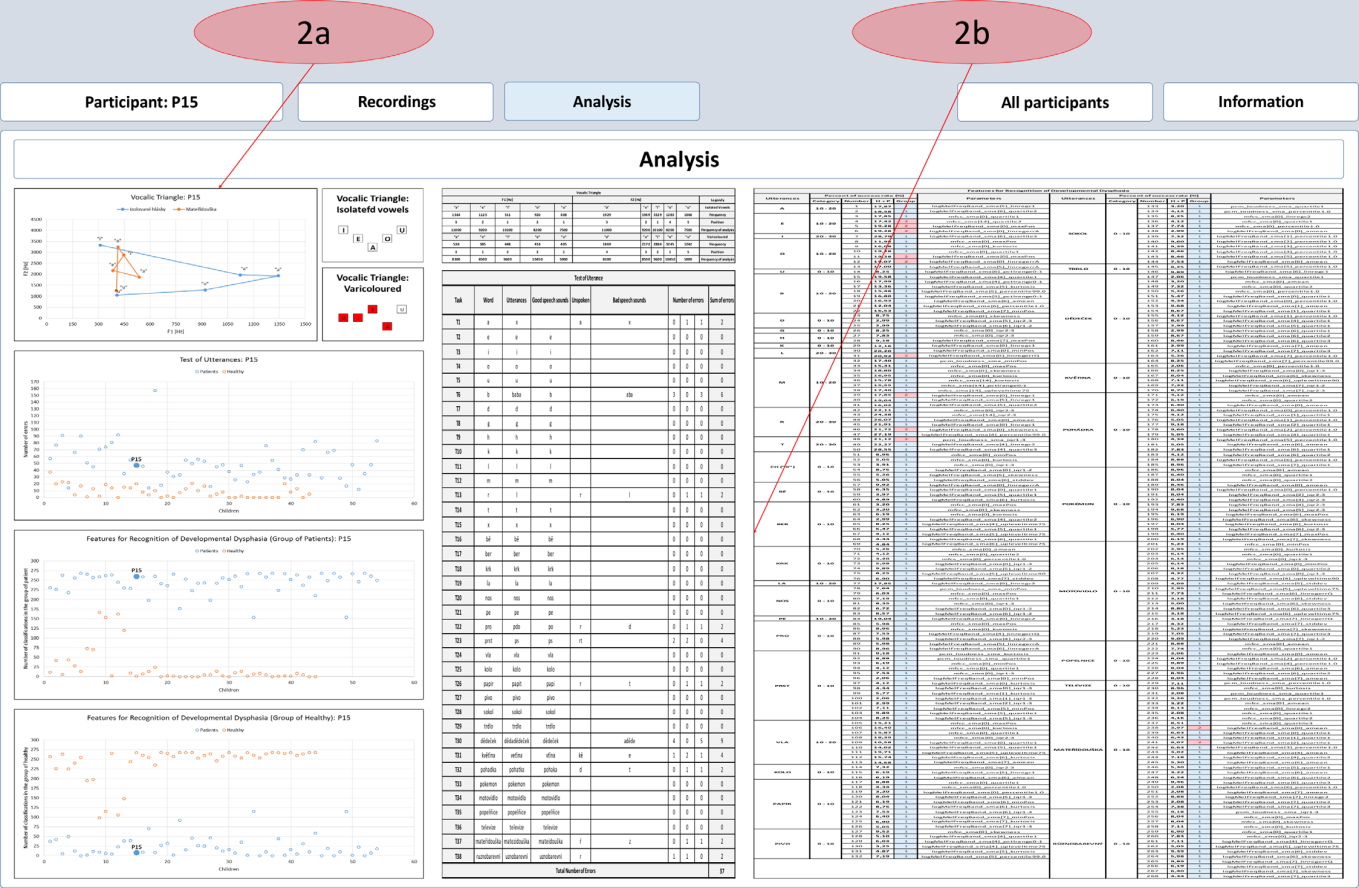
Common outputs of our analyses in the web tool. This figure shows the results from all analyses (Formants, Tests of Utterance, Extracting Features and Artificial Neural Networks); 2a) The panel shows all graphs; 2b) This section contains all the calculated values in the tables.

The basic goal for the development of the web tool was to ensure instant access to the results obtained from the research and to make management of the online speech database possible. The current web interface offers two main functionalities, and the information is divided into two sections to reflect this fact. The first is intended only for medical staff and researchers. Personal data protection is the reason for this approach. In this section, we present all the information about the speakers and the final results from the analyses used, including the calculated values, tables and graphs (shown in Figs 5 and 6). The second function of the web interface is intended for students. In the section corresponding to this function, students have the opportunity to listen to the selection of recordings (with unnamed personal data). They can also read the information about our methods and the procedures that we used in creating and managing the database and during computerized processing of the speech. Students can also download the selected recordings and use them as study material, as shown in Figs 5 and 6.

## Discussion

In this study, we described almost ten years of work on the collection of data comprising speech signals of children with or without specific language impairments. We focused not only on describing the entire children’s speech database and its subgroups but also devoted considerable efforts to describing the part of our methods that was used to classify cases, which was based on direct database processing.

The results of this research are a combination of many methods based not only on computerized processing of speech but also on medical diagnoses (e.g., EEG and MR tractography [21, 37]) via methods that are not discussed in this article. The computerized processing of normal and pathological children’s speech is also a combination of standard and nonstandard approaches. Using this combinatorial approach, we tried to eliminate weaknesses in these methods (formants are very sensitive to the quality of the recordings) and ensure correct classification using the strengths of the various methods (robustness of ANN to the quality of recordings). The combination of standard methods (formants analysis, MFCC, and so on), ANN methods (Supervised Self-Organizing Maps) and non-standard methods (number of errors in utterances) resulted in a high degree of successful classification for the cases. In addition, we introduced a real web application that is intended for medical staff and speech and language therapists, as well as for use as a means for students to become familiar with this issue.

### Speech database

The creation of a speech database designed for research focusing on children with specific language impairments (especially developmental dysphasia) is a very lengthy process. In our case, it represents almost ten years of constant work. In contrast to the other speech databases, data acquisition in this study was much more complicated because we worked with children, very often of preschool age, which can create specific problems. The most complex problem was how to maintain the children's attention throughout the recording. Children are very often distracted and may not focus on the recording: they may speak during the speech and language therapist’s speech, asking him about various things, or they may play with the microphone. Therefore, the recordings contain many unwanted sound remnants. Another problem was the reluctance of parents of children in both the controls and cases groups to agree to the research. The research process involved many examinations of the children, and parents had to fill out a medical questionnaire, which is one reason why the dataset is not larger. It is important to note that these are biological data with their own particular specificity, and the quantity of the data is not the most significant of their properties. A final problem was related to the technique and location of the recordings. The rooms were not soundproofed, and normal urban noises can be overheard in the recordings. The same applies to the quality of the recordings. At the beginning of our research study we had a SONY digital Dictaphone, and the recordings therefore had to be subsequently loaded onto a computer. This way of processing is not optimal because much background noise is included in the recordings. During the research, we exchanged the digital Dictaphone for a professional solution. In this method, the children’s speech is captured by means of a SHURE lapel microphone using tools by the company AVID (MBox–USB AD/DA converter and ProTools LE software) on an Apple laptop (iBook G4). These recordings were of high quality and contained almost no background noise. It is very complicated to compare recordings of low quality with those of high quality. Despite these problems and shortcomings, we managed to find several methods for identifying cases.

### Formants

Formant analysis provides information about the individual vowels in the frequency spectrum. Each vowel has a clearly defined location in the vocalic triangle when two conditions are fulfilled. First, the formants must be correctly classified. Second, the utterance must be properly spoken. The whole point of using formants in our research was to verify the correctness of the spoken utterances. Cases had problems with saying difficult (multisyllabic) words correctly. Formant analysis clearly verifies whether the vowels contained in utterances are pronounced correctly. If there are any errors in the analyzed vowel in the utterance, there is a shift in the frequency spectrum. This indicates that the speakers have the articulatory organs in a different position than the typical position and that the distribution of articulatory cavities has the wrong shape for a vowel. As a final result, these abnormalities lead to the malfunction of speech control in the brain. The disadvantages of using formant analysis include a notable susceptibility of the method to the quality of the recordings and the requirement for correct classification of the calculated formants.

### Tests of Utterance

A different approach, based on the number of errors in the utterances, was applied in the other method used. The advantage of this approach is that its function does not require complex computational methods, and it can be performed by anyone. This approach provides complete information about the most common errors and substitutions between speech sounds in the utterances made by the cases.

### Extracting Features

We focused on classification by the speech parameters that can be simply obtained and for which calculations can be performed without more complicated modifications of the speech signal. In total, we received 1,582 features for each utterance. We reduced the large amount of data obtained by applying correlations, and we selected the 30 features with the best results for the final evaluation. We obtained a 96.94% success rate for correct classification of the participants. The great advantage of this method lies in the small amount of adjustments made to the speech signal that was used.

### Artificial Neural Networks

The last selected method was ANNs, specifically Supervised Self-Organizing Maps. We used the SOM Laboratory program package. ANN functioning is based on the principle of biological neural networks. One indispensable advantage of the application of ANNs is their robustness in the setting of various artifacts contained in the speech signal and the minor requirements needed to improve the quality of the recordings. The very simple implementation of ANNs and the clear display of the classified data is a key feature, and these tasks are much more complex when using conventional statistical methods.

### Web Tool

We expect that the web application will be an interactive tool for researchers and students. The web tool directly connects the results of the research with education. Students can deepen their knowledge and can try various analyses on real data and compare them with our results. This application also reduces the work that is needed with the data for all persons working on research. Everyone has immediate access to the selected information of all speakers and to all the results of the analyses. Unlimited access to the database is reserved for medical staff and the other members of the research team. Cooperation between individual workplaces will be accelerated, and potential problems can be solved immediately.

## Conclusion

Our results prove that it is possible for cases to be clearly identified and distinguished from controls based on their speech and speech skills. We combined traditional and alternative approaches to this issue and obtained a resistance tool that is not dependent on the quality of the captured recordings. We found several different classification methods for cases. All these methods can be used separately for the classification of these children. Each method yields a high level of success in classifying cases, but each has its own particular limiting factors and shortcomings. Using these methods together, we were able to eliminate these shortcomings and obtain a powerful tool for diagnosing cases.

The possibility for classifying cases by an analysis of their speech is very significant for several reasons. The first is the opportunity to detect more children with this disorder. For these children, therapeutic treatment can be performed earlier. Another important reason is the financial aspect. Diagnostics that use speech are less demanding on hardware and are more financially feasible than other specific medical examinations.

Currently, we have resolved, in cooperation with the ethics committee, to allow access to the whole database to all researchers, clinicians and educators with an interest in this issue. The recordings and data will not contain any identifying information from participants. All recordings, data and applications used in this study are now saved and free to use on the server of The LINDAT/CLARIN Centre for Language Research Infrastructure (http://hdl.handle.net/11372/LRT-1597).
